# Reply to Flowers et al.: Existing thermochronologic data constrain Snowball glacial erosion below the Great Unconformity

**DOI:** 10.1073/pnas.2209946119

**Published:** 2022-08-22

**Authors:** Kalin T. McDannell, C. Brenhin Keller, William R. Guenthner, Peter K. Zeitler, David L. Shuster

**Affiliations:** ^a^Department of Earth Sciences, Dartmouth College, Hanover, NH 03755;; ^b^Department of Geology, University of Illinois at Urbana–Champaign, Urbana, IL 61801;; ^c^Department of Earth & Environmental Sciences, Lehigh University, Bethlehem, PA 18015;; ^d^Department of Earth & Planetary Science, University of California, Berkeley, CA 94720;; ^e^Noble Gas Thermochronology Laboratory, Berkeley Geochronology Center, Berkeley, CA 94709

The origin of the Great Unconformity has recently been debated (1–3). Flowers et al. (2) suggested that erosion of the Pikes Peak granite (Colorado) was caused by Neoproterozoic tectonism prior to the Cryogenian, and implied that this local signal, if correct, invalidated a Neoproterozoic glacial origin for the global phenomenon of the Great Unconformity (1). McDannell et al. (3) instead find that inversions of thermochronometric data from widespread North American locations and tectonic settings (including Pikes Peak) are consistent with a Cryogenian glacial contribution to development of the Great Unconformity. Here we address the Flowers et al. comment (4) on our work.

**Fig. 1.**
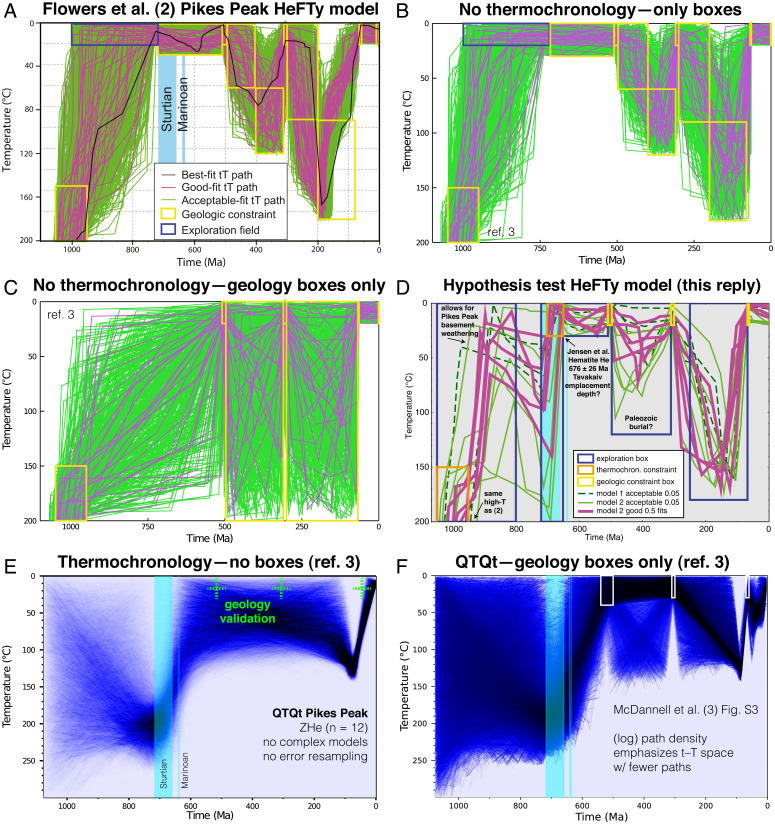
(*A*) Flowers et al. (2) HeFTy (9) model inverted “synthetic” dates from effective uranium binned/averaged observations (we do not condone this biased ad hoc approach). (*B*) Simple Monte Carlo model applying boxes only—without thermochronology data (3). (*C*) Model with Precambrian boxes removed; Cryogenian or earlier cooling allowed (3). (*D*) Three HeFTy models attempted here using 1) observed dates, 2) seven eU bins, and 3) five eU bins (2). Tavakaiv dike emplacement depth (10) and the timing of Pikes Peak granite weathering (2; 3) are interpretations. Our model actually tests *t*–*T* paths for both the tectonic and glacial hypotheses. Model #1 failed to generate any *t*–*T* paths; *P* value statistical tests fail for precise and/or high *n* data (11). Model #2 yielded few “acceptable” paths. Model #3 rapidly produced good-fitting solutions. Solutions are consistent with both the glacial and tectonic scenarios, yet better-fitting paths support heating and rapid exhumation during Snowballs. (*E*) QTQt model—simplest paths that best fit the observed Pikes Peak data (3). (*F*) QTQt model with only geologic constraints (3). Models demonstrate that it is rather a mistake to wield the limitations of inversion approaches ill-suited to deep-time problems just to generate favored thermal histories.

We fully agree regarding the merits of geologic information in constraining inversions. In fact, we state (3) that geologic knowledge should always be incorporated—whether as imposed constraints during inversion (5) or as holdout data for testing and validation (6). Critically, however, physical geologic constraints and interpretive assumptions are not equally valid (3) (Fig. 1). The latter, including any interpretation about which reasonable geologists may disagree, should not be heavily weighted or otherwise strictly imposed (2) in a modeling strategy.

Flowers et al. (4) incorrectly assert that we “show no data or metric to assess how well [our] preferred time–temperature (*t*–*T*) paths replicate the observations”; in fact, such data are shown in ref. 3, SI Appendix, figures S4–S12. We refrain only from selecting a best *t*–*T* path, since this would be misleading, due to inversion nonuniqueness. McDannell et al. (3) apply geologic constraints, and, unlike ref. 4, integrate all available chronometer data for inversions (ref. 3, SI Appendix, figures S1–S3). It is implausible that our “model outcomes are artifacts,” since we obtained results consistent with Cryogenian erosion for locations spanning thousands of kilometers, using search algorithm variants, different uncertainty estimates, and different types and quantities of input data.

McDannell et al. (3) explore *t*–*T* solutions constrained by 1) the data alone—providing an honest assessment of what can and cannot be resolved—and 2) the data plus reliable geologic constraints (either relaxed or omitted in cases of greater uncertainty). Deep-time chronometers modeled this way are truly assessing multiple hypotheses instead of simply imposing a preconceived interpretive model to which the data must conform (2). Such conformity is usually achieved only through excessive use of *t*–*T* “exploration boxes” and preferential data selection/averaging (e.g., refs. 2 and 7).

Is it more favorable to model all observed thermochronological data and independently address known uncertainties—or to preemptively reduce data quality and resolving power and thus universally require many *t*–*T* boxes to attain model convergence? Asserting that thermochronological data are “poor resolution” moves toward a paradigm where data complexity is ignored and inversions only fulfill the modeler’s preferred interpretation. The “Cryogenian cooling” forward-model paths in Flowers et al. (4) outperform their alternatives in reproducing overall data trends—consistent with the results of McDannell et al. (3). Finally, considering the geologic deficiencies of alternative exhumation mechanisms in the cratonic localities, Cryogenian glacial erosion remains the most parsimonious model (8).
